# Nanomaterial-Enhanced Microneedles: Emerging Therapies for Diabetes and Obesity

**DOI:** 10.3390/pharmaceutics16101344

**Published:** 2024-10-21

**Authors:** Mehrnaz Abbasi, Divine Afunu Boka, Hannah DeLoit

**Affiliations:** 1 Department of Nutritional Sciences, College of Human Sciences, Auburn University, Auburn, AL 36849, USA; 2 Department of Pre-Health Professional Curricula, College of Sciences and Mathematics, Auburn University, Auburn, AL 36849, USA

**Keywords:** nanomaterial, microneedle, drug delivery, diabetes, obesity, therapy

## Abstract

Drug delivery systems (DDS) have improved therapeutic agent administration by enhancing efficacy and patient compliance while minimizing side effects. They enable targeted delivery, controlled release, and improved bioavailability. Transdermal drug delivery systems (TDDS) offer non-invasive medication administration and have evolved to include methods such as chemical enhancers, iontophoresis, microneedles (MN), and nanocarriers. MN technology provides innovative solutions for chronic metabolic diseases like diabetes and obesity using various MN types. For diabetes management, MNs enable continuous glucose monitoring, diabetic wound healing, and painless insulin delivery. For obesity treatment, MNs provide sustained transdermal delivery of anti-obesity drugs or nanoparticles (NPs). Hybrid systems integrating wearable sensors and smart materials enhance treatment effectiveness and patient management. Nanotechnology has advanced drug delivery by integrating nano-scaled materials like liposomes and polymeric NPs with MNs. In diabetes management, glucose-responsive NPs facilitate smart insulin delivery. At the same time, lipid nanocarriers in dissolving MNs enable extended release for obesity treatment, enhancing drug stability and absorption for improved metabolic disorder therapies. DDS for obesity and diabetes are advancing toward personalized treatments using smart MN enhanced with nanomaterials. These innovative approaches can enhance patient outcomes through precise drug administration and real-time monitoring. However, widespread implementation faces challenges in ensuring biocompatibility, improving technologies, scaling production, and obtaining regulatory approval. This review will present recent advances in developing and applying nanomaterial-enhanced MNs for diabetes and obesity management while also discussing the challenges, limitations, and future perspectives of these innovative DDS.

## 1. Introduction to Drug Delivery Systems (DDS)

Different drugs can enter the human body through various anatomical routes, and selecting the appropriate delivery systems is crucial for achieving therapeutic goals [1]. To optimize the effects of therapeutic agents, DDS use various technologies to control their release and distribution within the body [2]. These systems manage drug administration via various oral, transdermal, pulmonary, mucosal, and intravenous pathways. They offer innovative methods to maximize therapeutic efficacy, improve patient compliance, and minimize side effects [3,4,5]. By precisely controlling the pharmacokinetics and pharmacodynamics of therapeutic agents, these systems enable accurate control over their concentration and distribution at target areas [5,6]. DDS has the significant capacity to overcome the drawbacks of traditional drug formulations, including their low solubility, limited bioavailability, and rapid clearance from the body. Advanced DDS can protect drugs from degradation, control release rates, and target specific tissues or cells, thereby enhancing therapeutic outcomes while reducing systemic toxicity [7,8,9]. DDS has additionally facilitated the development of innovative therapeutic strategies, such as targeted therapeutics, chronic illness sustained-release formulations, and non-invasive therapeutic agent delivery methods [7,10,11]. DDS are utilized in various therapeutic areas, including cancer, cardiovascular diseases, neurological disorders, infectious diseases, and metabolic diseases (e.g., diabetes and obesity). In oncology, for example, nanocarrier-based DDS have demonstrated potential in enhancing the effectiveness of chemotherapy while reducing unintended side effects [12,13,14]. Long-acting insulin formulations have greatly enhanced patient quality of life and glycemic control in diabetes treatment [15,16]. In addition, DDS development has been essential in extending the shelf life of current drugs and introducing novel therapeutic agents to the market, which would otherwise be challenging to administer effectively [3,4,7].

### 1.1. Conventional DDS

Conventional DDS, such as tablets, capsules, syrups, ointments, and injections, have been the primary methods of drug administration for years due to their simplicity and cost-effectiveness. However, they face significant challenges, including low bioavailability, inconsistent blood drug levels, and frequent dosing requirements [9,17,18]. These limitations often result in less effective therapeutic outcomes, increased side effects, and reduced patient compliance. Conventional DDS often lack mechanisms for sustained release, making it difficult to keep drug concentrations within the therapeutic range. Although they provide convenience and accurate dosing, they are limited by drawbacks such as poor absorption and lack of target specificity [7,9]. Advanced DDS, such as controlled [7,19,20] and stimuli-responsive [21,22], have emerged as significant advancements, offering improved therapeutic outcomes through better drug release and targeting control [9,19].

### 1.2. Transdermal DDS

Parenteral and oral are the two commonly used drug delivery methods. Since they are simple to use and enable patients to self-administer, small-molecule drugs are typically taken orally. However, therapeutic peptides and proteins are usually injected because they degrade rapidly in the stomach and have poor absorption. The need for more advanced delivery methods is apparent because, despite their effectiveness, injections are intrusive and poorly received by patients [23]. The transdermal drug delivery system (TDDS) presents an innovative approach to delivering therapeutic agents through the skin to achieve systemic effects [2,23]. These advanced pharmaceutical formulations offer several advantages over conventional DDS, including non-invasive administration, improved patient compliance, and avoidance of first-pass metabolism [2,24]. A typical TDDS consists of a drug reservoir, a rate-controlling membrane, and an adhesive layer. The drug penetrates various layers of the skin and permeates across the skin into the systemic circulation [25,26]. This mechanism lowers dosing frequency, enables controlled drug release, and maintains steady drug levels. The first FDA-approved transdermal patch for motion sickness was the scopolamine patch in 1979. Other notable examples include nicotine, fentanyl, testosterone, and estradiol patches [2,27,28,29]. Various enhancement techniques have been developed to overcome the skin’s barrier properties. These include chemical enhancers that temporarily disrupt the stratum corneum structure, iontophoresis (INT), which uses a small electric current to drive ionized drug molecules through the skin, and microneedle (MN), creating microchannels for drug delivery [24]. Recent developments include hydrogels and smart polymers that react to physiological changes, nanocarriers that improve drug stability and penetration, and 3D-printed patches for customized transdermal system fabrication [27,30,31]. These developments seek to increase current systems’ effectiveness and the number of drug options for transdermal delivery [2,32].

## 2. MN Technology

MN technology represents a significant advancement in TDDS, providing a minimally invasive method to deliver therapeutic agents through the skin [33,34]. MNs are micron-scale needles, typically ranging from 25 to 2000 μm in length. They penetrate the skin’s outer layer, the stratum corneum, without reaching the nerves, minimizing pain and discomfort [35]. There are different types of MNs, including solid, hollow, coated, and dissolving MNs, each with a specific fabrication process and mechanisms of action [36]. Solid MNs create microchannels in the skin to enhance permeability, while hollow MNs allow for the direct infusion of drugs. Coated MNs are covered with a drug-containing dispersion that dissolves upon insertion, and dissolving MNs are made from biodegradable polymers that dissolve completely, releasing the drug in a controlled manner [33,35]. Common MN fabrication methods include photolithography with selective etching, machining with chemical etching, machining with micro-milling, laser cutter, 3d printing, micro-injection molding, hot embossing, electrohydrodynamic atomization, laser machining, electric-discharge machining with laser machining, soft lithography, drawing lithography, centrifugal lithography, and droplet-borne air blowing [36,37]. One of the main advantages of MN technology over conventional drug delivery methods is its minimally invasive design, which significantly reduces the pain and discomfort often experienced with hypodermic needles [35,38]. This is especially beneficial for patients with needle phobia or those who need frequent injections, like diabetics. Furthermore, MNs improve patient compliance due to their ease of use and the ability to self-administer without professional assistance. The controlled release mechanism of MNs provides a consistent and sustained release of the drug, which enhances bioavailability and therapeutic effectiveness while reducing the need for frequent dosing [34,35,39]. MN technology offers various applications in drug delivery, effectively administering vaccines, insulin, and other therapeutics [35,40,41]. This method enhances immunization for vaccines like influenza, measles, and polio, providing a painless alternative to traditional injections [40]. In diabetes management, MN systems for insulin delivery improve glycemic control and patient compliance [42,43,44]. Additionally, MN technology has been studied for delivering analgesics such as lidocaine [45,46] and fentanyl [47,48], hormones like estradiol [49,50] and human growth hormone [51,52], anticancer drugs including 5-fluorouracil [53,54] and doxorubicin [55,56], antibiotics such as gentamicin [57,58], and biotherapeutics like monoclonal antibodies [59]. MN technology has a wide range of applications demonstrating its potential to improve therapeutic efficacy and increase the number of drugs that may be delivered transdermally [60]. Clinical trials and case studies have demonstrated the efficacy and safety of MNs, supporting their widespread adoption in medical practices [61,62].

## 3. Nanotechnology in Drug Delivery

Over the past few decades, nanotechnology has enabled DDS to overcome the limitations of traditional methods. By incorporating nanoscale materials, these advanced systems enhance therapeutic precision and effectiveness while minimizing side effects. Nanocarriers, which are nanoscale systems ranging in size from 1 to 1000 nm, play a crucial role in this advancement. They efficiently protect and deliver small-molecule therapeutic or imaging agents with high loading efficiency [63,64]. The unique properties of nanocarriers, such as their small size, large surface area, and ability to be targeted, have sparked significant interest in their development for treating multiple diseases. These features enable nanocarriers to optimize therapeutic efficacy, reduce side effects, and improve stability compared to conventional drug forms [65,66].

A diverse array of nanocarriers has been developed, including dendrimers, liposomes, solid lipid nanoparticles (NPs), polymersomes, polymer-drug conjugates, polymeric NPs, peptide NPs, micelles, nanoemulsions, nanospheres, nanocapsules, nanoshells, carbon nanotubes, and gold NPs, each with unique properties and advantages [65,66]. **Dendrimers** are nano-sized, hyperbranched molecules with a radially symmetric, well-defined, and homogeneous structure featuring tree-like arms or branches. They are used in drug delivery because they can encapsulate or bind to drugs, improving solubility and stability. Their ability to interact with drugs through both covalent and non-covalent bonds provides flexibility in delivery methods [67]. **Liposomes** are spherical vesicles composed of lipid bilayers capable of carrying both hydrophilic and hydrophobic drugs, enhancing bioavailability and reducing toxicity. They are the most widely used nanocarriers for active molecules due to their high biocompatibility, biodegradability, and low immunogenicity. Liposomes improve drug solubility and enable controlled distribution, with the ability to modify surfaces for targeted, prolonged, and sustained release. Over time, liposomes have evolved from conventional types to long-circulating, targeted, immune, stimuli-responsive, and actively targeted forms. Numerous liposomal drug delivery systems are clinically approved for treating diseases like cancer and infections, with many others in advanced clinical trial phases [68]. **Solid lipid NPs** are small lipid-based nanocarriers stabilized by emulsifiers, remaining solid at body temperature. They offer benefits such as drug protection, ease of production, biocompatibility, and biodegradability. However, their crystalline structure can lead to low drug loading efficiency and potential drug expulsion during storage, along with an initial burst release of drugs. Despite these drawbacks, solid lipid NPs are promising for enhancing drug stability and controlled release [69]. **Polymersomes** are synthetic vesicles formed by the self-assembly of amphiphilic block copolymers, which consist of hydrophobic and hydrophilic segments. They provide a stable environment for drug delivery by encapsulating hydrophilic molecules in their aqueous core and hydrophobic molecules within their membrane. The properties of polymersomes can be adjusted by modifying the block copolymer composition, making them suitable for applications in drug delivery, diagnostics, and bioimaging [70]. **Polymeric NPs**, made from synthetic or natural polymers, are biodegradable and enable targeted drug delivery. They are at the forefront of drug delivery designs due to their precise control over properties like size, shape, charge, and surface functionality. These NPs can navigate biological barriers to target specific sites, encapsulate diverse therapeutic agents, and release them in response to stimuli. Despite their advantages, their clinical application remains limited [71]. **Peptide NPs** are self-assembling structures that efficiently deliver therapeutic peptides and have versatile biomedical applications, including drug delivery, inhibiting biomolecular interactions, and molecular imaging [72]. **Micelles** are amphiphilic colloidal structures with 5 to 100 nm diameters that enhance hydrophobic drugs’ solubility by orienting their hydrophobic cores inward. Nano micelles are particularly effective for cancer therapy due to their high drug-loading capacity and ability to penetrate deep into tumor tissues, overcoming drug resistance through the enhanced permeation and retention effect. They can also encapsulate hydrophilic drugs when formed as reverse micelles. Micelles facilitate sustained drug release for chronic diseases and improve drug targeting, solubility, and stability, while their hydrophilic outer layer extends circulation time and supports active targeting [73]. **Nanoemulsions** are nano-sized emulsions consisting of oil droplets dispersed in water or vice versa, used to improve the delivery of poorly soluble drugs. They are thermodynamically stable systems formed by mixing two immiscible liquids into a single phase using surfactants and co-surfactants with nano-scale droplet sizes. Unlike regular emulsions, nanoemulsions have much smaller particle sizes. These colloidal systems act as drug carriers, enhancing therapeutic efficacy and reducing adverse effects. Their applications include treating infections of the reticuloendothelial system, enzyme replacement therapy in the liver, cancer treatment, and vaccination. Nanoemulsions can be oil-in-water, water-in-oil, or bi-continuous, and their small size makes them transparent. They can also incorporate magnetic NPs to improve site specificity [74,75]. **Nanospheres** are polymeric NPs with a matrix structure commonly used as drug carriers in clinical applications. They encapsulate or attach drugs, forming a homogeneous structure that protects against enzymatic and chemical degradation. Nanospheres can be biodegradable or non-biodegradable, including modified starch, albumin, and polylactic acid. They facilitate organ-targeted drug release and can be administered via oral, nasal, or parenteral routes. Advantages include their ability to pass through small capillaries, target specific organs, reduce toxicity, and lower dosage frequency. However, they can be difficult to handle, prone to aggregation, and subject to rapid clearance [76]. **Nanoshells**, especially gold nanoshells, hold great promise in biomedical applications like imaging, targeted therapy, gene delivery, tissue welding, and drug delivery systems, focusing on cancer imaging and treatment. They are safe, biocompatible, stable, and can attach to various therapeutic materials. Nanoshells are categorized into two main types: hollow nanoshells and core-shell nanoshells, both used in drug delivery, imaging, photothermal therapy, and studying microenvironments. Variants include silica nanoshells, polymeric nanoshells, antibody-conjugated nanoshells, and radiolabeled nanoshells [77]. **Nanotubes** are unique materials characterized by their hollow, tubular nanostructure with nanometer-scale diameters. They can be composed of single or multiple layers of atoms, forming a hollow core. Carbon nanotubes are particularly notable for their high target specificity and potential in drug delivery due to their high aspect ratio, flexible surface chemistry, and controllable structure. Carbon nanotubes can enter cells through various mechanisms, such as endocytosis and passive diffusion. However, their hydrophobic nature necessitates functionalization, achieved through covalent or non-covalent bonding, to improve their compatibility for drug delivery, including carrying nucleic acids and antimicrobial agents. Carbon nanotubes are classified into single-walled and multi-walled types, and they are extensively used in commercial applications like fuel cells, photovoltaics, and biomedicine. Despite their promise in medical applications, such as drug transport, tumor imaging, and photothermal therapy, Carbon nanotubes pose potential health risks like asbestos, including carcinogenicity and respiratory issues. Therefore, while Carbon nanotubes are promising for medical use, their safety and health implications require careful consideration [78,79]. **Gold NPs** are composed of gold atoms, known for their unique optical and electronic properties due to their nanoscale size. These properties make them valuable in various fields, especially biomedicine and technology. Gold NPs are used for drug delivery in medicine, enhancing targeted therapy and bioavailability. They are also employed in photothermal and photodynamic therapies to destroy cancer cells through localized heating when exposed to certain light wavelengths. In diagnostics, gold NPs improve imaging techniques like MRI and CT scans by enhancing contrast, and they are used in biosensors to detect biological molecules. Beyond biomedicine, they are used in catalysis, photovoltaics, and sensors due to their tunable properties. However, their development and application must address potential toxicity and environmental impact [80].

Despite the advantages of nanocarriers, several limitations are associated with their use in DDS. Ensuring safety and biocompatibility remains a significant challenge, as an ideal nanocarrier should be non-toxic, biodegradable, and capable of evading the immune system to prevent being recognized as foreign. Achieving these characteristics is complex and requires extensive research and testing. Additionally, scaling up production while maintaining consistent quality is difficult, as variations can lead to differences in efficacy and safety, compounded by the high cost of sophisticated manufacturing processes. Regulatory challenges also complicate clinical translation due to the lack of standardized assessment protocols; regulatory bodies require robust characterization methods, safety guidelines, and stability maintenance for approval. Furthermore, nanocarriers must remain stable in biological environments until they reach their target sites, as stability issues can compromise their effectiveness, leading to premature drug release or degradation. Another critical challenge is effectively targeting specific cells or tissues without off-target effects. Moreover, some nanocarriers suffer from short half-lives and low solubility, limiting their effectiveness in drug delivery applications. Despite these limitations, the field of nanocarrier-based drug delivery continues to advance, driven by innovations in materials science and nanotechnology. Ensuring the successful clinical application of nanocarriers requires addressing challenges related to safety, scalability, regulatory approval, and stability, and it is essential for continued research and collaboration to fully realize their potential in medicine [81,82,83].

### 3.1. Nanotechnology in TDD

Nanotechnology has significantly advanced TDDS by enhancing skin penetration, allowing controlled release, and boosting therapeutic effectiveness [84]. Various types of nanocarriers, such as liposomes, solid lipid NPs, polymeric NPs, and nanostructured lipid carriers, are used in TDD. These advanced nano-scale carriers help overcome the skin’s natural barrier and improve drug delivery. Each type of nanocarrier has unique properties that can be customized for specific drugs and treatment goals, allowing for a wider range of medications to be effectively delivered transdermally [85,86]. These nanocarriers have improved skin penetration due to their small size, leading to greater drug absorption for TDD [87]. Additionally, encapsulating drugs within nanocarriers helps protect them from degradation, extending their shelf life and efficacy [85]. Furthermore, nanocarriers can be engineered for controlled drug release, allowing for sustained therapeutic levels over longer periods [31,85]. They also enhance the solubility of poorly water-soluble drugs, improving their bioavailability. Certain nanocarriers can be modified to target skin structures or cell types, improving therapeutic effectiveness and minimizing side effects [88,89]. Recent advancements in nanotechnology have led to the development of stimuli-responsive nanocarriers, which can release drugs in response to external triggers like heat or pH changes [90,91,92]. Additionally, integrating MNs with nanocarriers has been explored to enhance skin penetration, offering a promising approach for transdermal drug delivery [93]. Nanocarrier-based TDDS face challenges in ensuring long-term safety, addressing potential skin irritation, and developing cost-effective large-scale manufacturing processes for successful clinical implementation [94].

### 3.2. Integration of Nanomaterials with MNs

Combining nanomaterials with MNs has transformed TDDS, significantly enhancing their efficacy and adaptability [95]. This novel integration overcomes the conventional limitations of MNs by using the unique characteristics of nanomaterials, producing a stronger and more efficient drug-delivery platform [64,96]. The nanoscale features enhance mechanical strength, increase drug loading capacity, and allow for precise control over drug release. This approach effectively addresses common challenges in TDD, such as limited skin penetration, inconsistent release profiles, short circulation time, and undesirable toxic effects. As a result, it expands the potential uses of MN systems for medical treatment [84,96,97]. Nanomaterials such as NPs, nanofibers, nanocomposites, nanowires, nanotubes, and nanocrystals significantly enhance the mechanical properties of MNs. This improvement makes them stronger, more flexible, and more deformation-resistant. Consequently, they can penetrate the skin more effectively and withstand mechanical stress more efficiently [98,99]. For instance, adding carbon nanotubes or graphene to MNs enhances their tensile strength, enabling deeper skin penetration [100,101]. Furthermore, nanomaterials allow for more precise control over drug release kinetics and greater drug loading capacities, resulting in sustained therapeutic levels over time [96]. Nanostructured lipid carriers and solid lipid NPs can enhance drug solubility and provide controlled release profiles [102,103]. Nanomaterials also improve transdermal drug permeation. Nanocrystals, for instance, increase skin deposition and enhance skin penetration compared to standard formulations [31]. However, challenges remain, including ensuring long-term safety, addressing potential skin irritation, and developing cost-effective manufacturing processes. Further research is needed to evaluate the safety of NP accumulation in the body and its long-term effects [94].

## 4. Applications of Nanomaterial-Enhanced MNs for Diabetes and Obesity Management

The advancement of innovative therapies and delivery methods offers significant potential for improving the management of metabolic disorders like diabetes and obesity [104,105], which have become widespread globally [106,107]. Current treatments often face challenges such as limited efficacy, poor targeting, and low patient adherence, leading to adverse side effects. MN enhanced with nanomaterials presents an innovative solution, combining targeted therapeutics and biosensing in a minimally invasive format [93,98,104]. These systems combine nanomaterials with painless transdermal delivery, facilitating continuous glucose monitoring, precise insulin administration, and effective anti-obesity drug delivery. By incorporating various nanomaterials, MNs enhance mechanical strength, increase drug-loading capacity, and improve responsiveness to physiological changes. This innovative approach can potentially enhance therapeutic outcomes, increase patient compliance, and enable personalized treatment strategies for chronic metabolic conditions. Research shows nanomaterial-enhanced MNs could transform diabetes and obesity treatment, improving patient quality of life [108,109,110]. Table 1, Table 2, Table 3 and Table 4 summarize recent studies on developing and applying nanomaterial-enhanced MN systems for managing diabetes and obesity. 

Recent advancements in glucose monitoring systems have led to the development of innovative MN-based technologies for real-time glucose measurement [110,111,112,113]. Li X et al. [113] developed an innovative swellable MN patch incorporating gold NPs for efficient interstitial fluid extraction and real-time glucose detection. This two-layered design enables rapid, minimally invasive sampling and colorimetric analysis, providing a stable, sensitive, and user-friendly alternative to conventional blood glucose monitoring methods in diabetes. Another approach involves NP-integrated hydrogel MN continuously monitoring glucose levels in dermal interstitial fluid. This method was developed by GhavamiNejad P et al. [111] and has shown high sensitivity and accuracy in diabetic rats, with a low detection limit and the ability to track glucose changes after insulin administration. Chen et al. [110] also developed an innovative implantable glucose biosensor using microporous Polyvinylidene Difluoride (PVDF) membranes sandwiched between nanomaterial layers. This device demonstrated excellent sensitivity, rapid response, and sustained biocompatibility during in vivo testing in mice. The sensor maintained its performance over 21 days, showing its potential for long-term, minimally invasive, and accurate continuous glucose monitoring. Another promising novel MN-based electrochemical glucose sensor has been developed by Y Yoon et al. [112], featuring a three-electrode system integrated into a silicon MN array. This technology demonstrates a linear response to glucose concentrations in the 3–20 mM range, with a sensitivity of 17.73 ± 3 μA/mM-cm^2^. The sensor’s design and performance suggest significant potential for painless and accurate glucose monitoring in diabetes management, offering a promising alternative to traditional testing methods (Table 1).

**Table 1:** Summary of recent studies on developing and applying nanomaterial-enhanced MN patches for glucose monitoring in diabetes.

Delivery Device	Therapeutic Agent/Drug and Application	Type and Characteristics of Nanomaterials and MN	Experimental Models	Results	Ref.
***Glucose monitoring***					
**Swellable colorimetric MN**	AuNPs with GOx-like activity/Glucose monitoring	**NP** PVA-AuNPs Size: 5.8 nm Zeta potential: −22 mV **MN** Dissolving 10 × 10 array, Hight (1000), and Base (500) μm	Glucose solutions and agarose gels Streptozotocin (STZ)-induced diabetic male Sprague-Dawley rats	Visible color change with increasing glucose levels High glucose selectivity over interfering substances Successful glucose detection in diabetic rats	[113]
**Hydrogel MN-continuous glucose meters**	No drug/Glucose monitoring	**NP** PEDOT: PSS Ag-Pt **MN** Dissolving 10 × 10 array, needle height (800), pitch (500), and base width (200) μm.	Porcine skin Agarose gels Streptozotocin (STZ)-induced diabetic male Sprague-Dawley rats	High sensitivity for ISF glucose in diabetic rats 0.9 mM detection limit, covers hypoglycemia Robust MNs for consistent skin penetration Enhanced ISF interaction via MN swelling Accurate post-insulin glucose tracking	[111]
**Nanomembranes** **coated MN**	GOx enzyme/Glucose monitoring	**Nanomembrane** Polyaniline nanofiber, platinum NPs, GOx enzyme, and porous layers Size: Micro-pores 3–5 µm Nafion 300 nm **MN** Solid A needle of 1800 μm height	PBS solution Sprague-Dawley rats.	High glucose selectivity, low interference Rapid response time Accurate in vivo glucose monitoring	[110]
**MN-based electrochemical sensor: multi-walled carbon nanotube forest and platinum NPs.**	No drug/Glucose monitoring	**Nanotube** Multi-Walled carbon nanotubes, Platinum (Pt) NPs Size: 50 to 100 nm **MN** Solid 15 × 15 array, Hight (380), and Base (500) μm	PBS solution	Linear response to glucose Low noise and stable performance	[112]

**Table 2:** Summary of recent studies on developing and applying nanomaterial-enhanced MN patches for insulin delivery in diabetes.

Delivery Device	Therapeutic Agent/Drug and Application	Type and Characteristics of Nanomaterials and MN	Experimental Models	Results	Ref.
***Insulin delivery***					
**Hollow MN (MicronJet600™)**	Proinsulin peptide C19-A3 conjugated to gold NPs/Diabetes	**NP** Gold NPs Size > 5 nm Covalently coupled to the proinsulin peptide C19-A3. **MN** MicronJet600™ 600 μm length	Diabetic individuals	Safe and well-tolerated No β-cell function decline in limited participants Stable C-peptide levels in some participants No significant changes in glycemic control markers	[114]
**Nanovesicles-loaded MN**	Glucosamine-modified Insulin/Diabetes	**Nanovesicles** Nanovesicles (including RBC vesicles bound with glucosamine-modified insulin Size: 200 nm Loading capacity of Glu-Insulin: 1 wt % **MN** Dissolving 15 × 15 array, Hight (600), and Base (300) μm	PBS solution Streptozotocin (STZ)-induced type 1 diabetic mouse model	Increased Glu-Insulin release from vesicles Sustained glucose control vs. subcutaneous insulin Delayed blood glucose rise Reduced hypoglycemia risk Lower hypoglycemia index than free insulin	[115]
**Polymeric NP-loaded MN**	Insulin/Diabetes	**NP** Polymeric NPs based on carboxymethyl chitosan and conjugated linoleic acid Size: 200 nm Loading capacity: 83.78 ± 3.73% **MN** Solid 36 arrays, Hight (75–150) μm	Franz diffusion cells (Rat skin) Streptozotocin (STZ)-induced diabetic male Sprague-Dawley rats	Increased insulin permeation Sustained glucose reduction in rats Long-lasting glucose control Painless, minimally invasive delivery	[116]
**Glucose-responsive gold nanocluster-loaded MN**	Insulin/Diabetes	**Nanocluster** Gold Nanoclusters Size: 11.1 ± 2.0 nm and 22.6 ± 2.0 nm for blank and loaded NPs Loading: 848, 951, and 1297 μmol of insulin per gram of gold **MN** Dissolving 11 × 11 array, Hight (755), and Base (290) μm	Streptozotocin (STZ)-induced type 1 diabetic mouse model	Glucose-dependent insulin release Effective blood glucose reduction in diabetic mice Mechanically strong for skin penetration and insulin delivery	[117]
**H_2_O_2_-responsive mesoporous silica NP-loaded MN**	Insulin, along with glucose oxidase/Diabetes	**NP** Mesoporous Silica NPs Size: 175 nm Zeta potential: −34.9 mV EE: 66.1% Loading capacity: 13.2% **MN** Dissolving 10×10 array, Hight (550), and Base (550) μm	Streptozotocin (STZ)-induced diabetic male Sprague-Dawley rats	Glucose and H_2_O_2_-responsive insulin release from MN GOx enhances insulin release in high glucose conditions Effective blood glucose reduction in diabetic rats GOx in MNs boosts insulin release and hypoglycemic effect Improved pharmacological and relative bioavailability	[118]
**Nanovesicles-loaded INT mediated MN**	Insulin/Diabetes	**Nanovesicles** Size: 91.0–107.4 nm. EE: 89.05 ± 0.91% Zeta Potential: +27.8 mV **MN** Solid 296 needles, Hight (800), and Base (260) μm **INT** 0.2 mA/cm^2^, 100 Hz frequency for 5 h	Guinea pig skin Streptozotocin (STZ)-induced diabetic male Sprague-Dawley rats	Highest permeation rate Increased in insulin permeation Significant blood glucose reduction in rats	[109]

**Table 3:** Summary of recent studies on developing and applying nanomaterial-enhanced MN patches for diabetic wounds.

Delivery Device	Therapeutic Agent/Drug and Application	Type and Characteristics of Nanomaterials and MN	Experimental Models	Results	Ref.
***Diabetic wounds***					
**Nanozyme-loaded MN**	Catalase-templated nanozyme and polymyxin B/Diabetic wounds	**Nanoenzyme** CAT-Mn (SH)x@PM Synthesize Catalase (CAT)-supported natural enzymes (CAT-Mn (SH)x) with polymyxin B immobilized on its surface Zeta potential: 4.21 mV **MN** Dissolving 20 × 20 array Hight (800), and Base (500) μm	HEK293 cells Escherichia coli Streptozotocin (STZ)-induced diabetic male Balb/c mice	Accelerated wound healing in diabetic mice. Reduced inflammation and faster wound closure Promoted angiogenesis Increased VEGF levels and blood vessel formation Facilitated M1 to M2 macrophage polarization Strong antibacterial activity against *E. coli*	[119]
**Nanocomposite-loaded MN**	CeO_2_ and Ag/Diabetic wounds	**Nanocomposite** Ag@MSN@CeO_2_ NP: CeO_2_ core, a mesoporous silica NP (MSN) shell, and an outermost layer doped with Ag. Size: 126.74 nm Loading capacity: 10.5% **MN** Dissolving 12 × 12 array, Hight (600), and Base (300) μm	Human fibroblasts (HFBs) Human umbilical vein endothelial cells (HUVECs) Sprague-Dawley rats	Strong antibacterial effect Decreased cellular ROS Enhanced antioxidant enzyme expression Accelerated wound healing and reduced scarring Improved tissue regeneration and reduced inflammation	[120]

**Table 4:** Summary of recent studies on developing and applying nanomaterial-enhanced MN patches for obesity.

Delivery Device	Therapeutic Agent/Drug and Application	Type and Characteristics of Nanomaterials and MN	Experimental Models	Results	Ref.
***Obesity***					
**Soluble rosiglitazone NP-loaded MN**	Rosiglitazone, Oleanolic acid/Obesity	**NP** Size: 102–156 nm Zeta potential: 11.7–18.9 mV PDI: <0.2 EE: >90% **MN** Dissolving 15 × 15 array, Hight (804), Width (210) and Base (700) μm	Franz cell (Mouse abdomen skin) C57BL/6 mice	Reduced body weight and fat Improved inflammation Promoted white adipocyte browning Decreased the mRNA levels of the proinflammatory factors TNF-α, IL-1β, and IL-6 Increased the mRNA level of the anti-inflammatory factor IL-10 in the iWAT Reduced blood levels of glucose, TG, Tchol, LDL	[121]
**Nanomicelles-loaded MN**	Tetradecanoic acid-2,4-dinitrophenol ester/Obesity	**Nanomicelles** Size: 215.94 nm Zeta potential: −14.43 mV **MN** Dissolving 10 × 10 array, Hight (600), and Base (300) μm	3T3-L1 preadipocytes C57BL/6 diet-induced obese mice	Enhanced endocytosis in preadipocytes Stronger lipid droplet reduction Increased UCP1 expression and browning Higher iWAT accumulation, lower off-target distribution Reduced systemic toxicity Decreased body weight and fat mass Improved lipid profile and metabolism	[108]

Chen Q et al. [114] introduced a glucose-responsive insulin delivery system using MN patches loaded with red blood cell vesicles or liposomes, effectively controlling blood glucose levels in diabetic mice without causing hypoglycemia. Similarly, Tatovic et al. [115] evaluated the safety and feasibility of intradermally administering proinsulin peptide conjugated to gold NPs via MN as an immunotherapy for type 1 diabetes, demonstrating safety and potential for enhanced tolerogenic effects. P. Zhang et al. [116] also combined carboxymethyl chitosan NPs with MN arrays to improve transdermal insulin delivery, significantly increasing insulin permeation rates. Furthermore, Zhang Y et al. [117] presented alginate-based MN patches integrated with glucose-responsive gold nanoclusters, which maintained normal blood glucose levels in diabetic mice for up to 2.8 days with a single application. Xu et al. [118] also developed an MN delivery system integrating H_2_O_2_-responsive mesoporous silica NPs loaded with insulin and glucose oxidase, demonstrating effective glucose-responsive insulin release. In another study, Chen H et al. [109] showed a transdermal insulin delivery method using charged unilamellar nanovesicles combined with MNs and INT, achieving a remarkable increase in permeation rates compared to passive diffusion. These approaches offer promising minimally invasive alternatives for diabetes treatment, potentially improving patient compliance and glycemic control through painless, effective, and user-friendly insulin delivery methods (Table 2).

Novel MN patches incorporating nanoenzymes and NPs have been introduced for enhanced diabetic wound healing. Cai et al. [119] created an MN patch incorporating nanozyme-supported natural enzymes and polymyxin B for treating diabetic wounds. This novel approach demonstrates sustained hydrogen sulfide release, enhanced enzyme and antioxidant activities, and effective antibacterial properties, promoting wound healing through dual anti-inflammatory and antibacterial effects and improved angiogenesis and nerve regeneration. Similarly, Yu et al. [120] designed a soluble MN patch loaded with multifunctional Ag@MSN@CeO_2_ NPs. These NPs include a cerium dioxide (CeO_2_) core with anti-inflammatory and antioxidant properties and a mesoporous silica (MSN) shell promoting angiogenesis. The patch showed deep-tissue penetration and offered multiple therapeutic benefits. These benefits include antibacterial activity, reduced reactive oxygen species (ROS), improved vascular regeneration, and enhanced collagen deposition. Both approaches significantly accelerated the healing process of diabetic wounds, providing promising solutions for this challenging condition (Table 3).

Chen et al. [121] developed an innovative rapid-dissolving MN patch containing rosiglitazone NPs for treating obesity. This method showed significant effectiveness in obese mice by effectively penetrating the skin and releasing the drug in the dermis. The treatment reduced body weight and fat, improved inflammation markers, and induced white adipocyte browning (converting white adipose tissue (WAT) to metabolically active brown/beige adipose tissue). Importantly, it lowered mRNA levels of proinflammatory factors (e.g., Tumor necrosis factor alpha (TNF-α), Interleukin 1 beta (IL-1β), and Interleukin 6 (IL-6)) while increasing the anti-inflammatory factor Interleukin 10 (IL-10) in inguinal WAT (iWAT). Additionally, the treatment decreased blood levels of glucose, triglycerides, total cholesterol, and LDL. Similarly, Liang et al. [108] developed an innovative MN patch delivery system for 2,4-dinitrophenol (DNP), utilizing tetradecanoic acid-DNP ester nanomicelles to enhance targeted delivery to adipose tissue. This approach demonstrated significant anti-obesity effects in a high-fat diet-fed mouse model while improving biosafety compared to oral administration. The system showed enhanced preadipocyte endocytosis, stronger lipid droplet reduction, and increased Uncoupling protein 1 (UCP1) expression and browning. Notably, it achieved higher accumulation in iWAT with lower off-target distribution, reducing systemic toxicity. The treatment decreased body weight and fat mass and improved lipid profile and metabolism. These findings, combined with other recent advancements in MN patch technology for obesity treatment, highlight the potential of minimally invasive, targeted drug delivery methods to enhance efficacy and reduce side effects compared to traditional administration routes, representing a promising approach for developing more effective and user-friendly obesity treatments (Table 4).

## 5. Challenges and Future Perspective

The development and application of nanomaterial-enhanced MNs for TDD face several challenges but also hold significant future potential, particularly in managing chronic diseases. One major challenge is creating cost-effective, large-scale production methods that ensure consistent quality and performance across batches. Regulatory barriers must be addressed, especially safety concerns related to nanomaterials and protocols for innovative technologies. Long-term stability is crucial for improving the shelf-life of nanodrugs in MNs while maintaining drug stability during storage and application. To ensure effective MN penetration across the skin, it is crucial to enhance their mechanical strength while considering the drug’s loading capacity and maintaining structural integrity. It is crucial to prioritize optimizing the drug release profile and developing delivery systems that respond to specific stimuli. These factors are key in enhancing the effectiveness of therapeutic interventions in managing chronic diseases [93,122]. Customizing MN designs, formulations for personalized treatments, and biosensors for real-time monitoring can significantly improve treatment outcomes.

## 6. Conclusions

The advancement of TDDS, especially MN technology enhanced with nanomaterials, has created new ways to manage chronic metabolic diseases like diabetes and obesity. These advanced systems offer targeted delivery, controlled release, and improved bioavailability of drugs. They also allow for continuous monitoring and personalized treatment strategies. Integrating nanotechnology with MNs has expanded the possibilities for smart, responsive drug delivery and real-time health monitoring. Despite significant progress and potential benefits, challenges persist regarding biocompatibility, scalability, and regulatory approval. MNs enhanced with nanomaterials and other advanced delivery methods can potentially improve chronic disease management, benefiting millions worldwide.

## Abbreviations

High-density lipoprotein (HDL), Inguinal white adipose tissue (iWAT), Low-density lipoprotein (LDL), Microneedle (MN), Nanoparticle (NP), Polydispersity index (PDI), Triglyceride (TG), Total cholesterol (Tchol), Uncoupling protein 1 (UCP1).
